# Using systems thinking in state health policymaking: an educational initiative

**DOI:** 10.1057/hs.2013.17

**Published:** 2014-01-17

**Authors:** Karen J Minyard, Rachel Ferencik, Mary Ann Phillips, Chris Soderquist

**Affiliations:** 1 Georgia State University, Atlanta, U.S.A.; 2 Pontifex Consulting, Hanover, New Hampshire, U.S.A.

**Keywords:** systems thinking, system dynamics, policymaking, legislators, education, health policy

## Abstract

In response to limited examples of opportunities for state policymakers to learn about and productively discuss the difficult, adaptive challenges of our health system, the Georgia Health Policy Center developed an educational initiative that applies systems thinking to health policymaking. We created the Legislative Health Policy Certificate Program – an in-depth, multi-session series for lawmakers and their staff – concentrating on building systems thinking competencies and health content knowledge by applying a range of systems thinking tools: behavior over time graphs, stock and flow maps, and a system dynamics-based learning lab (a simulatable model of childhood obesity). Legislators were taught to approach policy issues from the big picture, consider changing dynamics, and explore higher-leverage interventions to address Georgia's most intractable health challenges. Our aim was to determine how we could improve the policymaking process by providing a systems thinking-focused educational program for legislators. Over 3 years, the training program resulted in policymakers' who are able to think more broadly about difficult health issues. The program has yielded valuable insights into the design and delivery of policymaker education that could be applied to various disciplines outside the legislative process.

## Background

According to national health rankings, Georgia falls near the bottom for most health status indicators – 36th in the 2012 United Health Foundation's ‘America's Health Rankings' (2012). The complexities behind these poor rankings continue to challenge Georgia's legislators as they work to improve the health and well-being of their constituents.

Compositions of legislatures vary state by state. The Georgia legislature consists of 56 senators and 180 representatives. The legislative session begins in January and runs for 40 days. Only a few legislators have paid staff, but both houses have small research and budget offices. Currently, the Republican Party holds the majority in both chambers.

Similar to other states, each year Georgia legislators face an array of bills and proposals on specific health care issues. These bills typically call for small adjustments that chip away at costs, improve access to care, or address gaps in services. True and lasting change depends on a major shift from this current narrowly defined, issue-specific policy focus to a broader systems approach.

## Introduction

By any measure, policymaking at the national and state levels is a difficult, complex riddle that researchers and practitioners have been trying to understand for years. What factors impact decision makers' processes? What factors do they take into account when making difficult policy decisions? And how do they do it? In our investigation, the Georgia Health Policy Center (the Center), an applied research center at the Andrew Young School of Policy Studies at Georgia State University, discovered that most policy education for legislators addressed specific topics and was intended to support specific policy decisions or political positions (personal correspondence with Rose, Gehshan, Rich, Thomas & Tobbler, 2006). The literature tends to focus on the methods for conveying information and how policymakers take in and respond to information; little evidence exists that state legislators rely on university-based researchers for health policy analysis (Mooney, 1991; Coburn, 1998; Sorian & Baugh, 2002). Similarly, the experts described an emphasis on conducting specific processes (e.g., preparing succinct, but effective briefings and presentations for legislators; or conducting systematic reviews of health care literature) rather than engaging legislators to think about the impact of their policies on the whole system (Sorian & Baugh, 2002; Fox & Greenfield, 2006). In fact, even though the value of the collaborative use of dynamic systems tools is well documented in the literature (Richardson, 1996; Vennix *et al*, 1996; Hovmand *et al*, 2012), the Center staff found few examples of in-depth, proven approaches for engaging legislators in systemic policy educational programs, and very little evidence of programs to improve the way policymakers examine the tough, adaptive challenges in today's society. Over the last 7 years, in an attempt to understand and improve upon the policy process, the Center embarked on an educational initiative to provide Georgia legislators with a framework not only for understanding the reasons behind Georgia's poor health rankings, but also for changing the way they make decisions about health-related issues.

## Approach

In 2006, with a 3-year grant from the Robert W. Woodruff Foundation, the initiative began with a literature review. To learn more about the specific needs of Georgia's health policymakers, the Center conducted in-person, structured interviews with 10 leaders in the Georgia General Assembly. As in any state general assembly, Georgia legislators are expected to know ‘a little about a lot' of issues but can only become expert in a few policy areas. These areas often relate to their field of study or vocation, or a deep passion that they champion throughout their legislative careers. In this context, Center staff asked legislators for recommendations on how to teach the complexities of health care, what are the research needs of policymakers and how are they being met (or not), what are the best ways to translate and share relevant evidence and research findings with legislators, how to entice policymakers to attend educational sessions and keep them engaged, who should be included in the initiative, and how to keep it bipartisan. To a person, interviewees were positive about the initiative and willing to endorse the Center's efforts. Their insights were consistent and substantive, and led the Center to identify four broad categories of learners with regard to health policy.

*Group 1*: Novice legislators just beginning to learn about health and health financing and still struggling with the large number of unfamiliar terms and acronyms.

*Group 2*: Legislators interested in understanding complex health policy issues that are ‘hot' and controversial – but wanting the more concise version because they will soon be required to vote on a related bill.

*Group 3*: Legislators, often on health-related committees, who recognize the complexity and nuances of health policy and want to understand how the pieces of the system fit together.

*Group 4*: Legislators who hold positions of leadership and seek higher-level knowledge so that they recognize the implications of policy and resource decisions on health or how health fits into a broader context.

### Group 3: The Legislative Health Policy Certificate Program (LHPCP)

The LHPCP (the Certificate Program), the educational design for Group 3, is the flagship of this initiative. Created for state policymakers and legislative staff wanting a deeper understanding of health and health policy, the Certificate Program helped them develop the skills to approach policy issues as ‘systems thinkers', that is, to look at the big picture, consider multiple factors and their changing dynamics, and explore higher-leverage interventions to address Georgia's most intractable health challenges. We were interested in understanding if a systems thinking approach to legislative health policy education could begin to change the way legislators frame issues, ask questions, and consider solutions to complex health-care issues.

### Educational design: applying systems thinking to the curriculum

At its most basic level, systems thinking is concerned with connections between the components of a system – be it environmental, social, or political – and how those components relate to one another. It is a way of approaching a problem that utilizes multiple disciplines and critical thinking skills such as dynamic thinking (looking at a problem over time rather than as a single event), system-as-cause thinking (drawing the boundaries in such a way as to ensure the elements responsible for behavior – the causes – are included), and forest thinking (looking at the system from 30,000 feet above to see how things fit together) (Meadows, 2008; Richmond, 2010). The following section describes how systems thinking concepts were applied to the educational curriculum.

### The Six-Question Framework

In order to better operationalize aspects of systems thinking, the Six-Question Framework (Table 1) was developed by the Center in collaboration with a system dynamics modeler and facilitator. The Six Questions served as the foundation of the educational design and provided a construct for evaluating specific health content in the policy arena. The Certificate Program policymakers were not only asked to apply the Six Questions to various policy issues but were also encouraged to use them when tackling challenging health-related problems during the legislative session.

### Behavior over time graphs

**Figure 1:**
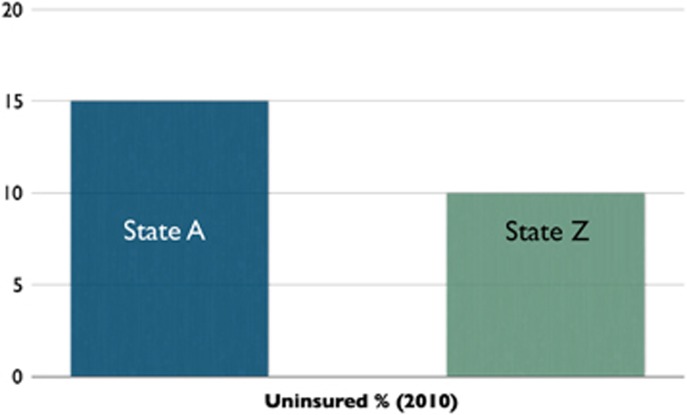
Example of a static graph.

**Figure 2:**
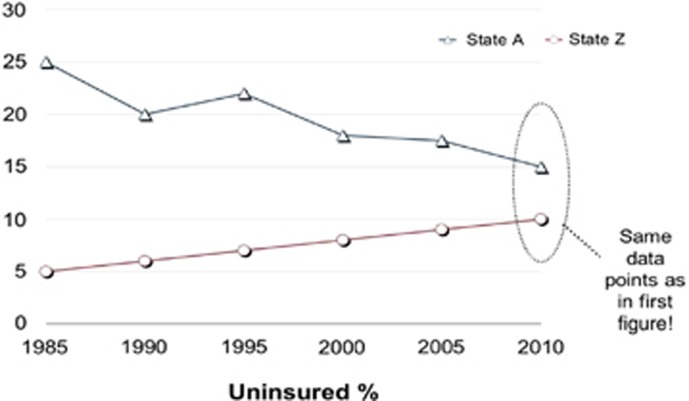
Example of a behavior over time graph.

Systems thinkers often use visuals to facilitate learning. A systems thinking tool that participants learned to use in the Certificate Program was behavior over time graphs. Legislators were often asked to draw behavior over time graphs where they think about an issue not just from a *single point in time* but rather as dynamically changing *over time*. Data at one point in time artificially narrows the boundary of the problem whereas expanding to longer term trends changes the nature of both framing a problem and thinking about solutions. For example, when looking at the graph in Figure 1, one might conclude that State A should reconsider its approach to the issue. They could learn a lot from State Z. However, by using a behavior over time graph (Figure 2) one would probably conclude that State A is doing a great job. They might even have something to teach State Z, who ought to be concerned.

### Stock and flow maps

**Figure 3:**
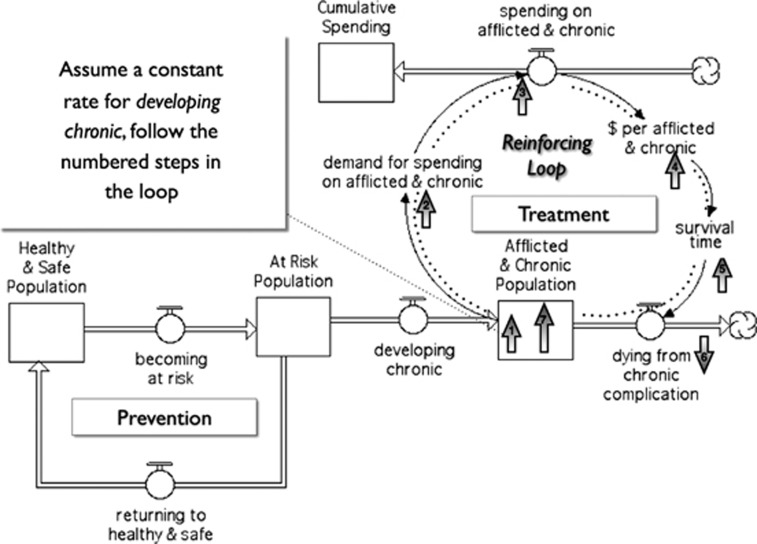
Stock and flow map.

Stock and flow maps are another tool of system thinkers. Stock and flow maps can depict a system in a common visual language and allow stakeholders to see how things are connected, where the boundaries of the system are, and how feedback loops contribute to the complex dynamics that occur in real-life situations. One such map (Figure 3) was generated by participants during the first session of the Certificate Program, and later refined, to facilitate conversation about disease prevention. The process of creating the map helped policymakers better understand the system in which this complex problem ‘lives', identify the levers for making change, and neutralize conflict or bias. Perhaps more importantly, its impact lasted well beyond the session by transforming how they framed this issue in subsequent policy meetings and dialog.

### System dynamics models

Another family of tools used to build systems thinking capacity is simulation models (e.g., learning labs, flight simulators, virtual worlds). Such tools have been shown to facilitate learning by allowing participants to explore the future and economic impact of specific policy changes on a selected problem (Vennix *et al*, 1996; Rouwette *et al*, 2002; Rouwette *et al*, 2011; Hovmand *et al*, 2012). In these ‘low cost laboratories', decision makers can conduct experiments, stop to reflect on results and then repeat under new conditions, and walk away better prepared for real-life scenarios (Sterman, 2006). In 2008, legislators in the Certificate Program chose childhood obesity as an issue to model because obesity among school-age children has tripled in recent decades – and Georgia is no exception. Reversing this complex epidemic requires a diverse set of policies and interventions, making it an ideal candidate for the systems thinking framework featured at the core of the Certificate Program. To test this idea, before the 2009 legislative session, the Georgia Health Policy Center convened 15 Georgia legislators and staff for half a day and gave each of them a laptop with a proprietary computer simulation based on system dynamics modeling. The simulation was designed by a collaborative team that included state legislators, legislative staff, and experts in nutrition, exercise physiology, epidemiology, economics, and system dynamics. It relied on epidemiological data and structure from a similar tool developed by the Centers for Disease Control and Prevention (Homer *et al*, 2006).

The simulation occurred in a real-time, hands-on learning lab environment. Participants were encouraged to express assumptions, predict outcomes, and inquire into differences between their expectations and the model's outcome. Following the simulation, participating legislators commented that the model informed their deliberations during the legislative session and contributed to the passage of a bill requiring fitness testing and stricter enforcement of physical education requirements in Georgia's school system.

Throughout the Certificate Program, the systems thinking skills were woven into the health topics taught to the participants. The Program consists of four ‘core' sessions and four issue-specific sessions. These eight sessions are delivered over the course of 4–9 months, depending on the legislative calendar. Core sessions covered the topics of health status, health financing, health coverage, and access, while issue-specific sessions were determined by the participants and ranged from childhood obesity to trauma care to health reform, depending on the requests of participants.

Early in the program, participants were introduced to six visuals that depicted the following concepts: the Six-Question Framework mentioned above; the health status in Georgia's 159 counties; the impact of health care on the local economy; the factors contributing to premature death; leverage points for intervening in the system; and the alignment of federal, state, and local resources. These visuals were consistently used throughout the eight sessions and became reference points to ground the discussion and explain complex issues.

#### The Advanced Health Policy Institute

Building on the success of the LHPCP, in 2012 the Center created the Advanced Health Policy Institute to refine legislators' skills in creating high-leverage solutions for adaptive health policy challenges. The 3-day course was open to those legislators and staff who had attended the Certificate Program. Legislators received additional content information related to financing and health reform, and learned to put forth their positions (their views, ideas, concerns, or suggestions) and the thinking that informs them, to respond productively to the inquiries of others, and to demonstrate active curiosity about alternate viewpoints – in the pursuit of deeper learning rather than full agreement (Weber, 2013). At the end of the Advanced Health Policy Institute, legislators were better able to understand and communicate the systemic causes of issues, the high-leverage ways to solve problems, and methods to have productive conversations and build support for implementation.

## Results

Thus far, 93 legislators and staff have participated in the Certificate Program; 64 of them are certificate holders. Nineteen legislators and staff have participated in the Advanced Health Policy Institute; 17 completed the entire training. Table 2 shows a breakdown of the number of participants awarded a certificate by year, type of attendee, and program.

Because of the unique nature of the Certificate Program and the Advanced Health Policy Institute, evaluation was an integral ongoing component of both programs. Legislators and staff completed a questionnaire at the end of each session. An example of one of the standard questions with the responses is provided in Table 3. The systems thinking approach was always highly received as indicated by participant responses provided in Table 4. After each session and at the end of each course, the evaluations were analyzed, and if necessary corrective actions were taken.

The Center staff was eager to understand whether the participants had begun to change the way they frame issues, ask questions, and consider solutions. Therefore, legislators and staff were asked to participate in structured individual and group interviews upon course completion. In response to an interview question about their most significant insights, comments ranged from the ‘6-Question Framework provided a well-rounded set of questions to set up an analytical framework' to ‘models show impact of change in a scientific way' to ‘I appreciate that we could drop our political personas and discuss ideas based on merits, which almost never occurs in the political arena'. In addition, Center staff attended many legislative committee meetings to ascertain whether the teachings from the sessions were having ‘real-world' application in policy discussions. While attending committee meetings did not reveal overt use of the systems thinking skills, individual legislators commented after the committee meetings that the systems skills prompted them to think about unintended consequences and how to integrate a variety of perspectives into the view of the system. One staff participant said he continued to use the concepts in the Six-Question Framework when tasked with supporting a tax policy committee of the legislature (personal communication with Betts, 2013).

Building the Certificate Program around a systems thinking framework was a distinctive approach to legislator education. While a gamble, it was a risk that paid off: legislators consistently give it high marks in their evaluations, and it has increased their capacity to engage in more fruitful, in-depth conversations. For example, using a system dynamics model proved to be very helpful in ‘raising the level' of the conversation regarding policies to address childhood obesity. Several months after interacting with the system dynamics model on childhood obesity, the General Assembly passed legislation on childhood obesity that had failed in previous years. Several attendees of our program commented that the level of conversation was different because of their experience with the model and impacted the passage of the legislation.

These educational sessions represent one of the few opportunities for bi-partisan, bi-chamber ‘get-togethers' when legislators can get to know one another and candidly discuss issues. As one legislator stated, ‘We may agree on the end goal, but have different ways to get there'. The value of such dialog was noted by Evan Bayh, as he explained the reasons for his retirement from the United States Senate in an Op-Ed article published in *The New York Times*: ‘Many good people serve in Congress. They are patriotic, hard-working and devoted to the public good as they see it, but the institutional and cultural impediments to change frustrate the intentions of these well-meaning people as rarely before …. Any improvement must begin by changing the personal chemistry among senators. More interaction in a non-adversarial atmosphere would help …. Listening to one another, absent the posturing and public talking points, could only promote greater understanding, which is necessary to real progress' (*The New York Times*, 2010).

The systems thinking skills taught in the Advanced Health Policy Institute provided an additional opportunity to help legislators frame the critical issues while the conversational capacity skills helped them talk and listen in ways that keep people engaged, rather than elicit usual defensive routines. The Center is continuing to explore how to reinforce these skills in other educational opportunities for state policymakers.

The systems thinking framework used in the course helped legislators with the ability to think more broadly about health, ask questions, and have discussions that help them better understand the issues and their solutions. We learned that participants wanted more – more health content but also more opportunities to meet in small groups and discuss challenging issues with their colleagues. The challenges facing our policymakers were not going to be solved by only learning about health. They needed new and different skills to help them collaborate with others who often approach issues differently than they do. There were no simple solutions that they could be taught to address these challenging issues. What they needed were systems thinking skills to help them better understand the issues, and communicate and collaborate with colleagues in ways that facilitate understanding.

## Conclusions and recommendations

We embarked on this endeavor eager to better understand and improve upon the policymaking process and attempt to change the way decision making is done on health issues. The process used by the Center to deliver this educational initiative has yielded valuable insights for the design and delivery of policymaker education, and could even be applied to various disciplines outside the legislative process.

Overall, participants were enthusiastically receptive to systems thinking, including the Six-Question Framework, stock and flow maps, and simulation models. Many of their comments have been used to refine the Certificate Program, design the Advanced Health Policy Institute, and bolster the educational process and impact. Some of the feedback confirmed what the Center already knew, some of it was new, and much of it has revealed the complexity involved in educating a part-time legislature, with few paid staff, trying to make policy in an economically disadvantaged state.

In addition to the systems thinking approach to legislator education, there were several programmatic lessons that may help others who embark on similar initiatives. They are:

*One size does not fit all*: When it comes to building knowledge among legislators, it is not realistic to expect to educate all legislators the same way with the same information. Categorizing policymakers into groups of learners allowed tailoring of educational strategies to meet specific needs. The Certificate Program and the Advanced Health Policy Institute were one categorization of learners: legislators, often on health-related committees, who recognize the complexity and nuances of health policy and want to understand how the pieces of the puzzle fit together. This category appeared most amenable to systems thinking. We continue to work with these other categories of legislators (Groups 1, 2, and 4) who often want different information and have different needs that can successfully be addressed in more traditional and less intensive ways.

*Secure endorsement of legislative leadership*: To help guide the initiative, the Center formed a Legislative Advisory Team that included the chairs of the health committees in the Georgia House and Senate, as well as the appropriations subcommittees on health. The support of these individuals was invaluable. They encouraged their committee members' participation, served as advisors in the content development, and authorized travel compensation and per diems to their members for attending the meetings. Their input was sought early on, in the initial design stage, and through regular ‘check-ins' at periodic intervals.

*Offer local, state, and national perspectives*: The Center expected legislators to be interested in health policy only as it affected their own local districts, or at best the state of Georgia, but the legislators were curious about issues and challenges that other states experienced as well. They inquired about other states' health rankings and what factors elevated those states to the top of those lists, what made Georgia different or the same, and what lessons could be learned from other states' experiences and successes.

*Meet participants' needs*: Although the Center prescribed the four core sessions of the program based on advice from the Legislative Advisory Team, half of the sessions were determined by participants. This made the content directly relevant to their interests and needs.

*Remain nonpartisan and neutral:* It is not often that people interact with legislators in a neutral unbiased approach without a political agenda. However, the Center has a long history of facilitating diverse audiences in difficult conversations and has developed strong relationships over the years with legislators by providing unbiased, evidence-based information for formulating health policy. This spirit of nonpartisanship was upheld in this initiative as well. In the design and delivery of educational sessions, the Center was conscientious in engaging outside experts from the metro Atlanta area and Georgia State University faculty in economics and health policy that had solid reputations for both quality and neutrality.

*Strive for a balance between course length and rigor*: The 3 h time block for each of the eight sessions was longer than recommended by interviewed experts and legislators. They cautioned that legislators would not make time for long sessions and should be expected to ‘come and go' as other pressing business took priority. While risky, the Center made a strategic decision that 3 h was the minimum amount of time needed to deliver the content and cover it with sufficient academic rigor and integrity. The wisdom of this decision was confirmed by participants' overwhelmingly positive feedback.

With House elections every 2 years, membership and leadership change regularly. New relationship building and identification of issues is an ongoing process.

With continued funding from the foundation, the Certificate Program will be offered again in 2013. The Center will continue to work with course alumni and newly elected leaders to better understand their needs and to design enhancements and systems thinking skills to support learners in all four groups.

## Figures and Tables

**Table 1:** A Six-Question Framework for evaluating health policy


1 What is the important (perhaps troublesome) trend related to health in Georgia? What is the shape of this trend over the past several years? 2 Who are the stakeholders concerned with the trend? 3 Why this trend (what is the cause, who is responsible)? 4 Where is there leverage (some policy) to address the underlying cause of the trend? 5 How will it work? How will it play out over time? How might unintended consequences occur? How might the policy positively or negatively impact a Health status? b State health spending? c Health care system? d Health equity? 6 When would the policy create an impact on health status? When would you see an improvement in some other indicators (i.e., spending, services)?

**Table 2:** Attendees of the LHPCP and the Advanced Health Policy Institute, 2008–2012

	*LHPCP*			*Advanced*	*Total*
	*2008*	*2009*	*2011*	*2012*	
Representatives	18	17	10	14	59
Senators	6	3	3	1	13
Legislative staff	6	7	4	4	21
Total	30	27	17	19	93

Attendance at all four core sessions and at least two issue-specific sessions were required to earn a certificate from the program.

**Table 3:** 2011 Certificate Program Session 4: sample responses from evaluations

*What were the most useful elements of this training?*	
1	Everything was useful
2	Data to support legislation; knowledge of health trends and challenges; models
3	Modeling of cause and effect on health trends
4	The modeling of how actions affect solutions
5	Feel a better grasp of the health exchanges
6	Working through models
7	Understanding of health reform

**Table 4:** Sample question from final session evaluations, 2008 and 2009

*What was the most beneficial aspect of the program? (Could check all that apply)*			
	*2008 (number of responses)*	*2009(number of responses)*	*Total*
Health policy content	11	10	21
Handout material	10	6	16
Small group exercises	2	4	6
Systems thinking approach	10	9	19
Conversations with colleagues	6	8	14
